# Role of HDAC3 on p53 Expression and Apoptosis in T Cells of Patients with Multiple Sclerosis

**DOI:** 10.1371/journal.pone.0016795

**Published:** 2011-02-08

**Authors:** Fanglin Zhang, Yaping Shi, Lily Wang, Subramaniam Sriram

**Affiliations:** 1 Department of Neurology, Multiple Sclerosis Research Center, Vanderbilt University Medical Center, Nashville, Tennessee, United States of America; 2 Department of Biostatistics, Vanderbilt University Medical Center, Nashville, Tennessee, United States of America; St. Georges University of London, United Kingdom

## Abstract

**Background:**

Histone deacetylase 3 (HDAC3) belongs to a family of proteins which plays an important role in protein acetylation, chromatin remodeling and transcription of genes, including those that are involved in cell proliferation and cell death. While increased expression of HDAC3 is seen in neoplastic cells, the role of HDAC3 in T cells and their role in autoimmune disease is not known.

**Methodology/Principal Findings:**

Applying Affymetrix GeneChip Human Gene 1.0 ST Array and the mixed effects model for gene set analysis, we compared gene expression profiles between multiple sclerosis (MS) patients and healthy controls (HC). Within the Apoptosis_GO gene set, the constitutive expression level of HDAC3 in peripheral blood mononuclear cell (PBMC) was significantly increased in MS patients when compared to controls. Following addition of trichostatin A (TSA), an inhibitor of HDAC3, we examined the expression of p53 by flow cytometry and p53 targeted genes by real time RT-PCR in MS and HC. Culture of PBMC with TSA resulted in increased expression of p53 in HC but not in MS patients. TSA treated T cells from MS patients also showed reduced sensitivity to apoptosis when compared to HC, which was independent of activation of p53 targeted pro-apoptotic genes.

**Conclusion/Significance:**

MS patients, when compared to controls, show an increased expression of HDAC3 and relative resistance to TSA induced apoptosis in T cells. Increased expression of HDAC3 in PBMC of MS patients may render putative autoreactive lymphocytes resistance to apoptosis and thereby contribute to autoimmunity.

## Introduction

Multiple sclerosis (MS) is an inflammatory demyelinating disease of the central nervous system (CNS) and is the most common neurological disease of the CNS affecting young adults [1]. Although the etiology of the disease is not known, the consensus of opinion is that MS is an autoimmune disease that is initiated by exposure to an environmental agent, most likely a pathogen, in genetically susceptible individuals [1], [2], [3], [4]. Expansion of autoreactive cells to putative neural antigens is thought to play a role in MS [5], [6]. Although a number of genes have been implicated in the development of MS, there are as yet no genes or gene sets that have been identified to be specific for MS.

One of the key events that regulate gene activation involves the architectural organization of the nucleosome that is mediated by the state of acetylation and deacetylation of lysine residues in the amino terminal tail of histones. Histone deacetylase (HDAC) leads to condensation of nucleosome, while histone acetyl transferase (HAT), leads to hyperacetylation and relaxation of the nucleosome thereby permitting the binding of transcription factors to the DNA thus allowing gene transcription [7], [8], [9], [10]. Acetylation and deacetylation of histone proteins play a pivotal role in the epigenetic regulation in many cell types, including immune lymphocytes. In addition to histones, HAT and HDAC use a number of non-histone proteins as substrates, including p53, NF-κb and STAT [11], [12], [13]. These protein substrates are involved in the regulation of cellular immunity and cell proliferation. Advances in the understanding of HDAC and HAT have provided new insights into the regulation of proteins that are involved in cell cycle progression, transcription and cell death [14], [15], [16].

HDACs are evolutionarily conserved proteins and are widely expressed. To date, at least 11 isoforms of HDAC are known in human but the functions of the different individual HDAC isoforms in regulating cell proliferation and cell death is not fully established. The different isoforms of HDAC are broadly grouped into four classes, based on their homology to yeast genes. Class I HDAC's (1,2,3 and 8) share homology with the yeast transcriptional regulator RPD3, class II HDAC (HDAC 4–7, 9–11) have similarity to yeast Hda1 gene and class III (SIRTs 1–7) share homology with NAD family of sirtuin proteins [9], [17]. HDAC3 contains an unusual C terminus and, unlike the predominantly nuclear HDAC1 and HDAC2, localizes to the nucleus, cytoplasm, and plasma membrane, indicating that HDAC3 is functionally distinct from other members of its class [9].

In some neoplastic diseases, expression and activation of p53 is regulated by HDAC [10], [18], [19]. P53 is a key transcription factorthat regulates the expression of genes involved in apoptosis and cell cycle arrest [20], [21], [22]. In the cytosol p53 binds to mdm2 and is rapidly degraded. Hence, the half life of p53 is short (less than 30 minutes) and constitutive expression of p53 is barely detectable in lymphocytes using conventional western blotting method. Acetylation of lysine residues on p53 abrogates the complex formation between p53 and mdm2, which will lead to an increase in the half life of p53 [12], [16]. Given that autoimmune disease represents unchecked expansion of autoreactive cells, we proposed that HDAC could regulate the expression and function of p53, and thereby affect the function of T cells, a key player in autoimmunity. We examined the constitutive expression of HDAC3 in peripheral blood mononuclear cell (PBMC) of patients with MS and compared them with HC. We also examined the effect of TSA, a known HDAC inhibitor, on the expression and apoptotic function of p53 in T cells of MS patients and HC.

## Results

### Microarray analysis of expression of the apoptosis gene sets between MS patients and healthy controls

**Figure 1:**
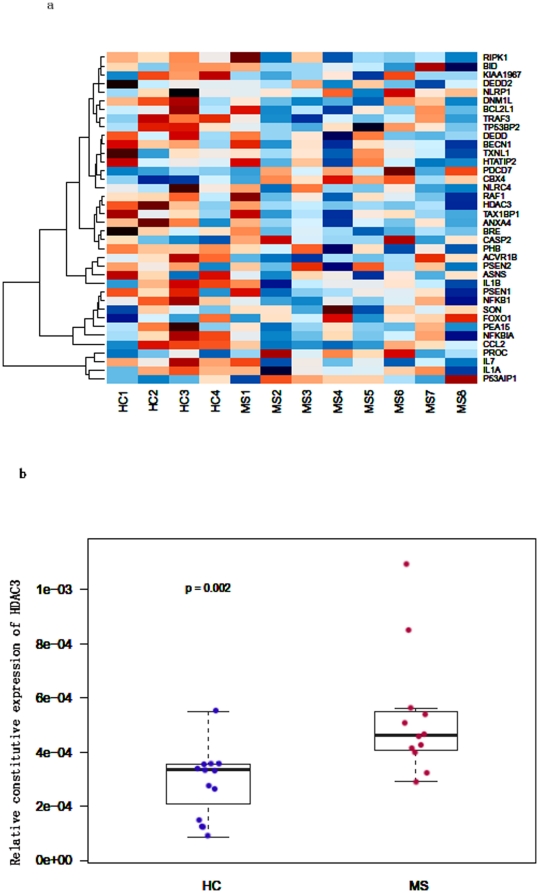
Constitutive expression levels of HDAC3 in HC and MS patients. 1a. Heat map shows constitutive expression level of genes involved in Apoptosis_GO gene set in MS patients and HC. The rows correspond to normalized, log (base 2) transformed gene expressions from PBMC of 4 HC and 8 MS patients. Red color indicates high expression and blue color indicates low expression. 1b. Real Time RT-PCR verified that the constitutive expression of HDAC3 in PBMC were significantly different (p = 0.002) in HC and MS patients. In these box plots, the bottom, middle and top of the box represent 25^th^, 50^th^ and 75^th^ percentile of gene expression levels normalized to beta actin in PBMC from 12 HC and 12 MS patients.

We used a unified mixed effects model for gene set analysis to screen 825 gene sets corresponding to the biological process categories from the Gene Ontology project [23]. Using this mixed model gene set analysis and by combining weak signals of differential expression across genes within the same gene set, we identified gene sets that were differentially expressed between MS and healthy controls in unstimulated PBMC. Table 1 shows that 8 out of 825 gene sets whose function related to cell death were down-regulated in MS patients. We examined the genes in the Apoptosis_GO gene set which differentiated MS from HC at a statistically significant level (Table 2 and Figure 1a). Of the 38 differentially expressed genes in the Apoptosis_GO gene set, HDAC3 was expressed at levels that were significantly higher in MS patients when compared to HC (p = 0.001). To confirm our observation from our micro-array studies, we quantitated the mRNA levels of HDAC3 in MS patients (n = 12) and controls (n = 12) using real time RT-PCR. As shown in Figure 1b, the expression of HDAC3 normalized to beta actin was higher in MS patients when compared to controls (p = 0.002).

**Table 1:** Differentially expressed gene sets involved in apoptosis biological process in MS patients compared to healthy controls.

*GO term*	*Gene set name*	*Size*	*Raw_p*	*FDR_p*
GO:0006915	APOPTOSIS_GO	424	0.0038	0.0689
GO:0012501	PROGRAMMED_CELL_DEATH	425	0.0041	0.0703
GO:0042981	REGULATION_OF_APOPTOSIS	335	0.0069	0.0961
GO:0043067	REGULATION_OF_PROGRAMMED_CELL_DEATH	336	0.0076	0.1023
GO:0006281	DNA_REPAIR	121	0.0322	0.2742
GO:0008624	INDUCTION_OF_APOPTOSIS_BY_EXTRACELLULAR_SIGNALS	27	0.0337	0.2833
GO:0006974	RESPONSE_TO_DNA_DAMAGE_STIMULUS	156	0.0425	0.3297

Mixed Models Analysis of the gene sets based on biological process categories from the Gene Ontology project. Size  =  number of genes in the gene set; Raw_p  =  nominal p-value; FDR_p  =  FDR adjusted p-value.

**Table 2:** Genes within the apoptosis gene set that were differentially expressed between MS patients and controls.

Gene	GenBank accession number	Direction of changed regulation	P-value	Description
HDAC3	NM_003883	up	0.0011	Histone deacetylase 3
NFKB1	NM_003998	up	0.0063	Nuclear factor NF-kappa-B p105 subunit
CCL2	NM_002982	up	0.0063	C-C motif chemokine 2 Precursor
IL1B	NM_000576	up	0.0069	Interleukin-1 beta Precursor
HTATIP2	NM_001098520	up	0.0070	Oxidoreductase HTATIP2
NLRC4	NM_021209	up	0.0073	NLR family CARD domain-containing protein 4
PHB	NM_002634	up	0.0116	Prohibitin
ACVR1B	NM_004302	up	0.0133	Activin receptor type-1B Precursor
NLRP1	NM_001033053	down	0.0135	NACHT, LRR and PYD domains-containing protein 1
PSEN1	NM_000021	up	0.0137	Presenilin-1
TXNL1	NM_004786	up	0.0138	Thioredoxin-like protein 1
CASP2	NM_032982	down	0.0161	Caspase-2 Precursor
P53AIP1	NM_022112	down	0.0165	tumor protein p53 regulated apoptosis inducing protein 1
BID	NM_001196	up	0.0195	BH3-interacting domain death agonist
IL1A	NM_000575	up	0.0200	Interleukin-1 alpha Precursor
NFKBIA	NM_020529	up	0.0213	NF-kappa-B inhibitor alpha
BECN1	NM_003766	up	0.0218	Beclin-1
PEA15	NM_003768	up	0.0241	Astrocytic phosphoprotein PEA-15
PSEN2	NM_000447	up	0.0244	Presenilin-2
RIPK1	NM_003804	up	0.0244	Receptor-interacting serine/threonine-protein kinase 1
TRAF3	NM_003300	up	0.0248	TNF receptor-associated factor 3
ANXA4	NM_001153	up	0.0276	Annexin A4
CBX4	NM_003655	down	0.0282	E3 SUMO-protein ligase CBX4
FOXO1	NM_002015	down	0.0294	Forkhead box protein O1
SON	NM_032195	down	0.0298	SON protein
PROC	NM_000312	down	0.0304	Vitamin K-dependent protein C Precursor
RAF1	NM_002880	up	0.0307	RAF proto-oncogene serine/threonine-protein kinase
DNM1L	NM_005690	up	0.0309	Dynamin-1-like protein
BRE	NM_004899	up	0.0313	Protein BRE
KIAA1967	NM_021174	up	0.0325	Protein KIAA1967
PDCD7	NM_005707	down	0.0333	Programmed cell death protein 7
BCL2L1	NM_001191	up	0.0380	Apoptosis regulator Bcl-X
DEDD2	NM_133328	up	0.0419	DNA-binding death effector domain-containing protein 2
TAX1BP1	NM_001079864	up	0.0433	Tax1-binding protein 1
TP53BP2	NM_001031685	up	0.0462	Apoptosis-stimulating of p53 protein 2
DEDD	NM_001039711	up	0.0476	Death effector domain-containing protein
IL7	NM_000880	up	0.0480	Interleukin-7 Precursor
ASNS	NM_001673	up	0.0493	Asparagine synthetase [glutamine-hydrolyzing]

Direction of changed regulation  =  the constitutive expression level of genes in PBMC from MS patients compared to that of healthy control.

### Activation of p53 by TSA in PBMC – dose and time kinetics in healthy controls

**Figure 2:**
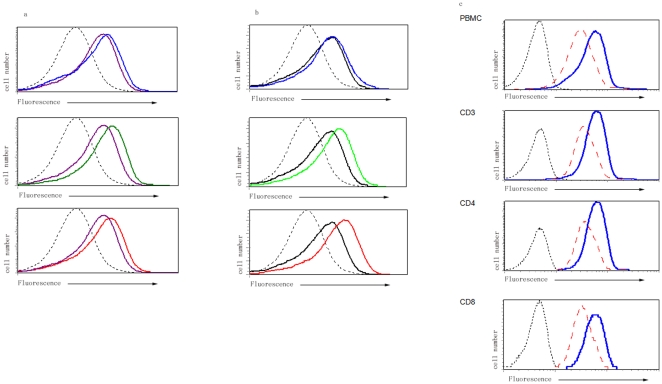
p53 expression in PBMC following culture with TSA in HC examined by flow cytometry. 2a. The expression of p53 following culture with TSA for 48 hrs at doses of 0.25 µM (top panel, blue line), 0.5 µM (middle panel, green line), 1.0 µM (bottom panel, red line). In all these three panels, the dotted line represents isotype antibody control, and the purple line represents the expression of p53 in lymphocytes cultured with DMSO in the absence of TSA. 2b. Increased expression of p53 following culture with 0.5 µM TSA at 6 h (top panel, blue line), 24 h (middle panel, green line) and 48 h (bottom panel, red line) respectively. In all these three panels, the dotted line represents isotype antibody control, and the black line represents expression of p53 in cells cultured with DMSO only. 2c. Expression of p53 in T cell subsets cultured with 0.5 µM of TSA for 48 h. Panels from top to bottom are: PBMC, CD3+, CD4+/CD3+ and CD8+/CD3+ subset of T lymphocytes. In all these four panels, the dotted line represents isotype antibody control; the red line represents the expression of p53 in cells cultured with DMSO in the absence of TSA and the blue line represents the expression of p53 in lymphocytes cultured with 0.5 µM of TSA for 48 h. The x axis represents increasing fluorescence intensity of p53, y axis represents cell number. Data are representative of 6 independent experiments.

Since HDACs are known to play a role in the expression of pro-apoptotic proteins including p53, we examined the expression of p53, under conditions that decreased HDAC3 expression. We predicted that trichostatin A (TSA), which inhibits HDAC, will increase expression of p53 in T lymphocytes. PBMC (1×10^6^ cells/ml) were cultured in vitro for 48h in the presence or absence of TSA, and the amount of p53 was determined by flow cytometry. We chose to use flow cytometry than conventional western blotting, since the former appeared to be more sensitive in detecting small differences in the cellular expression of p53. As shown in figure 2a and 2b, a dose and time dependant increase in p53 expression was seen following addition of TSA. P53 expression as determined by flow cytometry was maximal at a TSA concentration of 0.5 µM. After a 2 day culture, increased expression of p53 expression was seen in CD3+, CD3+/CD4+ and CD3+/CD8+ subsets of T lymphocytes (Figure 2c).

### Induction of apoptosis in PBMC cells cultured with TSA

**Figure 3:**
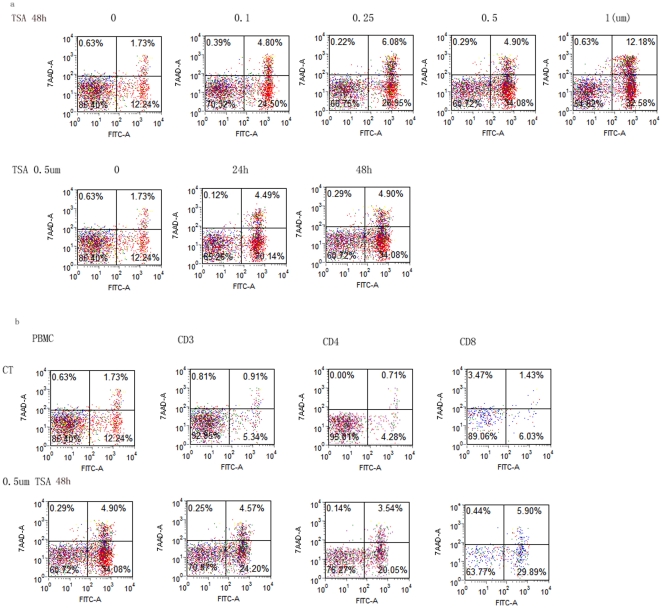
Detection of apoptosis by TSA in HC using flow cytometry. 3a. Top panel shows the increase in apoptosis in the presence of TSA at concentration of 0 µM, 1 µM, 0.25 µM, 0.5 µM and 1 µM after 48 h culture. Bottom panel shows the kinetic of apoptosis in PBMC treated with TSA at concentration of 0.5 µM for 6 h, 24 h and 48 h. 3b. Top panel shows apoptosis in DMSO treated cells as control. Bottom panel shows apoptosis in 0.5 µM TSA treated cells for 48 h. Both panels, from left to right, represent PBMC, CD3+, CD4+/CD3+ and CD8+/CD3+ lymphocytes respectively. Data are the representative of 15 independent experiments.

We next examined if increased expression of p53 in T lymphocytes in the presence of TSA leads to increase in apoptosis. As shown in figure 3a, there was dose dependant increase in the number of Annexin V+ cells and Annexin V+/7-AAD+ cells following the culture of PBMC with TSA. The number of viable PBMC decreased from 85.4% in the absence of TSA to 54.8% following a 2 day culture with 1 µM of TSA. Kinetic study performed with 0.5 µM of TSA showed that the number of viable cells, decreased from 85.4% at 0 h to 60.7% at 48 h (Figure 3a). Subset analysis of PBMC showed that the decrease in cell viability involved both CD3+/CD4+ and CD3+/CD8+ cells (Figure 3b).

### Induction of p53 in PBMC of MS patients and healthy controls

**Figure 4:**
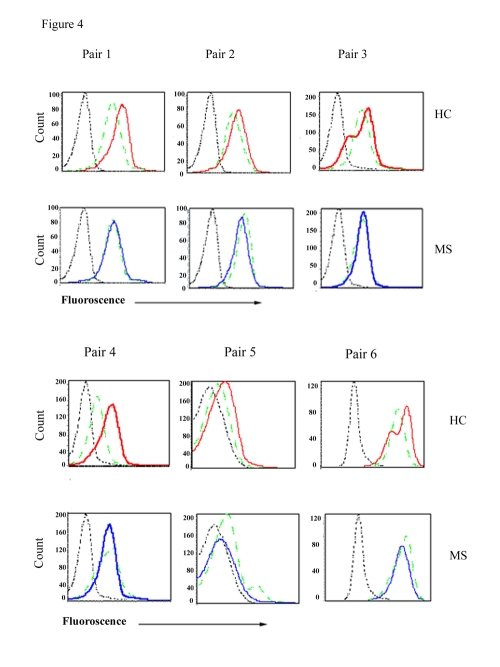
Detection of p53 expression in PBMC treated with TSA in HC and MS patients using flow cytometry. Increased expression of p53 in PBMC from HC but not in MS patients, treated with 0.5 µM TSA for 48 h. Dotted black line represents isotype antibody control; dotted green line, represents p53 expression in the absence of TSA; red line, p53 expression in the presence of TSA. Results are shown as paired samples of MS patients and HC; each pair was analyzed using flow cytometry on the same day.

Since MS patients showed an increase in the constitutive levels of HDAC3, we predicted that PBMC from MS patients will be more resistant to the inhibitory actions of TSA on HDAC and will be reflected in reduced levels of p53 in MS patients when compared to HC. In 6 MS patients and an equal number of HC, the expression of p53 in T lymphocytes cultured with TSA was examined. As shown in figure 4a, induction of p53 following culture of PBMC with TSA was seen in all 6 healthy controls. In contrast, we did not observe an increase in the expression of p53, following culture with TSA in any of the 6 MS patients (Figure 4).

### TSA mediated apoptosis is decreased in MS patients when compared to controls

**Figure 5:**
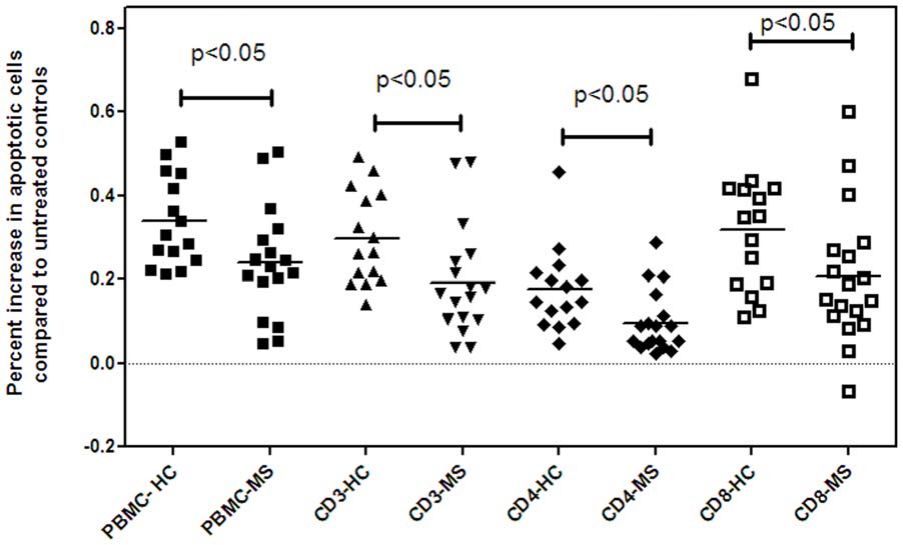
Susceptibility of PBMC to TSA induced apoptosis in HC and MS patients. Reduced susceptibility to TSA induced apoptosis in 18 MS patients when compared to 15 HC. PBMC were treated with 0.5 µM TSA for 48 h. Cells treated with DMSO in the absence of TSA served as a negative control.

Since PBMC of MS patients cultured with TSA failed to show an increase in cellular expression of p53, we predicted that PBMC of MS patients will be less susceptible to TSA induced apoptosis when compared to healthy controls. Apoptosis to TSA was examined in 18 MS patients and 12 HC and is shown in Figure 5. In whole PBMC as well as in CD3+/CD4+ and CD3+/CD8+ cells, MS patients showed reduced susceptibility to TSA mediated apoptosis (p<0.05 HC *vs.* MS). Since the MS population included some patients who were on immunomodulatory therapy, we examined if sensitivity to apoptosis was different between MS patients who were untreated (7) when compared to those on either beta interferon(5), glatiramer acetate (3), methotrexate (2), or mycophenalate (1). There was no significant difference between treated and untreated patients in the response of PBMC to apoptosis following culture with TSA, which may be due to the small number of patients in each treatment group (data not shown).

### Activation of p53 targeted genes in TSA treated PBMC in MS and HC

**Figure 6:**
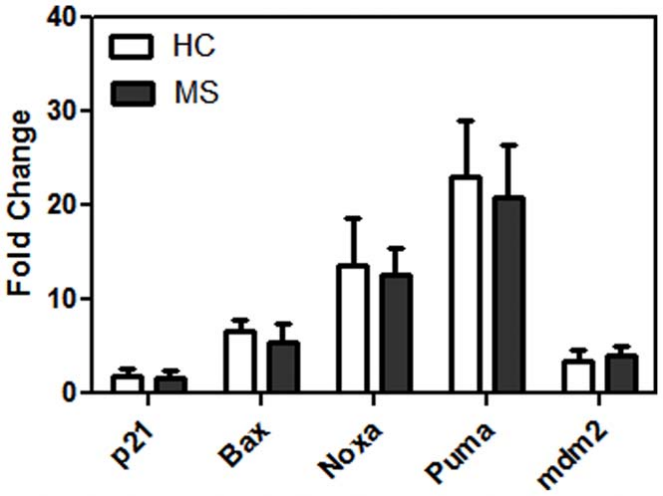
Real time RT-PCR studies on a panel of p53 regulated genes in PBMC treated with TSA between HC and MS. PBMC were treated with 0.5 µM TSA for 24 h, with DMSO in the absence of TSA as control. Results are expressed as fold change in expression of mRNA levels for each gene normalized to beta actin and compared to control.

To evaluate if the increased expression of p53 and the attendant increase in apoptosis seen in TSA treated PBMC was due to the transcriptional regulation of p53 regulated pro-apoptotic genes, we examined the expression of the following genes by real time RT-PCR: (i) p21, (ii) Bax, (iii) Noxa, (iv) Puma and (v) mdm2. As shown in figure 6, although there was a significant increase in the expression of all the examined genes in TSA treated PBMC (p<0.05 for all the five genes), there was no significant difference in the induction for these genes between MS and HC.

## Discussion

Our study shows that MS patients, when compared to controls, have an increased expression of HDAC3 and reduced susceptibility to TSA induced apoptosis in T lymphocytes, which was associated with a lack of an increase in p53 expression in MS. Our initial analysis based on the mixed effects model showed among 8 apoptosis related gene sets, Apoptosis_GO was the most significant gene set, differentiating MS from HC. Although there were 424 genes in the Apoptosis_GO gene set, we focused our study on HDAC3 since it showed the greatest difference in expression between MS and HC. While there have been a number of studies that have examined the expression pattern of genes in MS patients and compared it with HC, an increase in HDAC3 has not been previously reported [24], [25], [26]. Our study suggests that increased expression of HDAC3 in PBMC of MS patients might represent a new and as yet unrecognized biomarker for MS.

Since acetylation of histone and non histone proteins plays a key role in epigenetic regulation, we examined the effect of HDAC inhibition on key cellular function that is regulated by HDAC. TSA is a well known inhibitor of HDACs and maintains the sustained acetylation of proteins [10], [14], [27], [28]. The mechanism by which TSA induces cell death is not fully known, although studies in tumor cell lines have suggested increase in the expression of proteins involved in the DNA damage pathway, including the activation of p53 which may influence cell survival [29], [30].

Abnormalities in the regulation of HDAC3 expression have been associated with tumorigenesis. Over expression of HDAC3 has been seen in colon cancer cell lines [31], [32], and strong expression of HDAC3 correlates with a poor prognosis in patients with adenocarcinoma of the lung [33]. In acute lymphoblastic leukaemia patients, higher than median expression levels of HDAC3 were associated with a significantly lower 5-year event-free survival [34]. For these reasons, HDAC inhibitors are emerging as a new class of anticancer agent [18], [19], [32], [35]. These independent observations in tumor cells underscore the fact that over expression of HDAC3 can lead to cellular effects that affect cell growth, proliferation and the expression signaling proteins that regulate the function of key cytokines involved in innate immunity. At present factors that mediate the expression and activation of HDACs including HADAC3 remain speculative. The increased expression of HDAC2 in colorectal tumors is thought to be mediated by the activation of Wnt/beta-catenin pathway presumably through the activation of c-Myc [36]. In experimental inflammatory conditions such as systemic sepsis, glucocorticoids inhibit the expression of HDAC6 and HDAC3 [37]. It is not clear if in inflammatory immune disorders, expression of HDAC is influenced by pro and anti inflammatory molecules.

When compared to studies in cancer, expression of HDAC3 and its role in autoimmune disease has received limited attention. Synovial fibroblasts from patient with rheumatoid arthritis have shown increased expression of HDAC1 when compared to those with osteoarthritis. Inhibition of HDAC1 resulted in increased expression of pro-apoptotic proteins, including p53 and reduced proliferation of synoviocytes [38]. In experimental allergic encephalitis (EAE), a mouse model of MS, treatment of mice with TSA improved clinical scores of paralysis [39]. Improvement in disease scores was thought to be due to decrease in the number of Th1 encephalitogenic cells. In addition, TSA was shown to up-regulate anti–excitotoxicity and pro-neuronal growth and differentiation factors, resulting in improved neurological outcomes in mice with EAE [39]. While a general propensity for autoimmune syndromes in individuals over-expressing HDAC in immune cells may be anticipated, the factors underlying the development of organ specific autoimmunity is unknown.

The tumor suppressor protein p53 is a highly regulated transcription factor and is fundamental to the development of tumors. The major function of p53 resides in its ability to act as an important transcription factor of genes such as p21, Bax, Noxa, Puma and mdm2 which are involved in cell proliferation and death. It is now clear that in addition to its function as a transcription factor of pro-apoptotic genes, post translational modifications of p53 also plays a direct role in mitochondrial mediated apoptosis [40], [41]. The post translational modifications of p53 include acetylation, methylation, sumoylation and ubiquitination [42]. HDACs are known to regulate acetylation of p53 at residues Lys-320/Lys- 373/Lys-382or Lys-120 [43], [44]. The acetylation of p53 leads to the decreased degradation, thereby increases the constitutive level of the protein [12], [16]. Acetylated p53 can also directly translocate to the mitochondria, inhibiting anti apoptotic proteins BCL-1 and BCL-XL leading to its permeabilization and ultimate cell death [44], [45], [46].

Contrary to our expectation, the lack of an increase in p53 in PBMC of MS patients when compared to HC in the presence of TSA, was not associated with a parallel decrease in the transcription of p53 upregulated apoptotic genes. Induction of p21, Bax, Puma, Noxa and mdm2 by TSA were similar in MS to those in HC. Hence, the reduced sensitivity to the apoptosis of T lymphocytes treated with TSA in MS patients is unlikely to be due to decreased induction of p53 regulated pro-apoptotic genes. Acetylated p53 is more stable in T lymphocytes and we also speculate that the increase in acetylated p53 by TSA may lead to transcription-independent mitochondrial directed apoptosis, in a manner similar to the observations in tumor cell lines [44], [45], [46].

Our study suggests that increased constitutive expression of HDAC3 is associated with reduced apoptotic T lymphocytes in MS patients. While our study has focused mainly on T cells, the role of HDAC in other immune cells, including B cells, macrophages and NK cells needs to be addressed. Tumor cells appear to be sensitive to the inhibitory actions of TSA on HDAC. A number of selective and non selective HDAC inhibitors have been developed in the hope that decrease in HDAC activity would lead to activation of key proteins involved in regulation of cell proliferation and cell death [11], [47], [48], [49]. It is conceivable that HDAC inhibitors could also become part of the therapeutic strategy in the treatment of immune disorders including MS.

## Materials and Methods

### Subjects

34 patients with clinically definite MS (Table 3) and 33 healthy volunteers were recruited from the MS clinic of Vanderbilt Medical Center. The clinical subtypes of these MS patients were as follows: (a) 26 relapsing remitting MS (RRMS), (b) 5 secondary progressive MS (SPMS) and (c) 3 primary progressive MS (PPMS). Of the 26 RRMS, 14 were receiving beta IFN, 4 were on glatiramer acetate and 8 were on no therapy. Of the 8 patients with progressive MS, 5 were on no therapy, 2 had received methotrexate and 1 was on mycophenalate. The studies were approved by the Committee for the Protection of Human Subjects of the Vanderbilt University Institutional Review Board and the written consent forms were obtained from all participants involved in the study.

**Table 3:** Demographics of the patients with multiple sclerosis.

	MS subtype	gender	age
1	PP	F	38
2	RR	M	36
3	RR	F	53
4	RR	M	27
5	RR	F	35
6	RR	F	57
7	RR	F	35
8	RR	F	36
9	RR	F	45
10	RR	M	60
11	RR	F	49
12	RR	F	37
13	RR	M	55
14	RR	F	60
15	RR	F	49
16	SP	M	63
17	RR	F	44
18	SP	F	59
19	RR	F	57
20	PP	F	59
21	SP	F	21
22	RR	F	37
23	RR	F	38
24	RR	F	33
25	RR	F	54
26	RR	F	27
27	SP	M	55
28	PP	M	48
29	RR	F	42
30	SP	F	50
31	RR	M	51
32	RR	M	53
33	RR	F	55
34	RR	F	54

PP = progressive-relapsing multiple sclerosis; RR = relapsing-remitting multiple sclerosis; SP = secondary-progressive multiple sclerosis. F = female;M = male.

### Reagents and Chemicals

Antibodies: anti-CD4-FITC, CD4-Pacific blue, CD8-APC, CD3-PE and CD3-Pacific blue (BD Biosciences Pharmingen, San Jose, CA); anti p53-PE (Santa Cruz Biotechnology, Santa Cruz, CA).

Chemicals: Trichostatin A (TSA), dimethylsulfoxide (DMSO) and Ficoll-Hypaque (Sigma-Aldrich, St. Louis, MO).

The following kits were used: Annexin V-FITC & 7-AAD apoptosis detection kit (BD Biosciences Pharmingen, San Jose, CA); Fixation and permeabilization kit (Invitrogen, Carlsbad, CA); RNA isolation kit and RNase-free DNase set (Qiagen, Valencia, CA); Reverse Transcription kit (Applied Biosystems, Foster City, CA); iQ SYBR green supermix (Bio-Rad Laboratories, Hercules, CA); Agilent's Bioanalyzer microfluidic assay kit (Agilent Technologies, Palo Alto CA).

### Isolation and culture of PBMC

Peripheral blood mononuclear cells (PBMC) were isolated by density gradient centrifugation with Ficoll-Hypaque from freshly heparinized blood. The cells were washed in PBS and re-suspended at 1×10^6^ cells/ml in complete medium RPMI-1640 containing 2 mmol glutamine, 100 U/ml penicillin, 100 µg/ml streptomycin and 10% fetal bovine serum (Invitrogen, Carlsbad, CA). PBMC were cultured in the presence of TSA with the different concentration and time points as indicated in the results and figure legends.

### Total RNA extraction and reverse transcription

Total RNA was extracted from PBMC by RNeasy mini kit and treated with RNase-free DNase set, following manufacturer's recommendation. Agilent's Bioanalyzer microfluidic assay was applied to test RNA integrity. Spectrophotometric and fluorometric methods were combined to quantitate RNA. cDNA was generated from RNA using Reverse Transcription kit. 1 µg of total RNA was reverse transcribed in a total volume of 25 µl using 100 units of reverse transcriptase, 2.5 µl 10Χ RT buffer, 2.5 µl 10X random primer and 1.5 µl of 20 U/µl RNase inhibitor. The mixture was incubated for 10 min at 25°C, 120 min at 37°C, 5 seconds at 85°C and rapid cooling on ice. The cDNA samples were stored at –20°C.

### Real-time quantitative RT- PCR

Real-time quantitative RT-PCR reaction was carried out in an iCycler detection system in a 25 ul volume. The reaction mixture consisted of 12.5 ul iQSYBR Green Supermix, 200 nM of each primer, and 1 ul of cDNA template. Reactions were performed for 45 cycles (95°C for 15 s, 60°C for 30 s and 72°C for 30 s) after an initial 3 min incubation at 95°C. HDAC3 primers: 5′gctggagggaaaaggagtgg 3′and 5′ ggccttgggagagagaggaa 3′. Other primers for the target genes amplified are shown in the reference [50]. All reactions were done in triplicates. Values for each gene were normalized by those of internal control beta-actin using the Ct (threshold cycle) method and the fold changes compared to culture controls were calculated.

### Microarray analysis

Microarray experiments were carried out as described previously [50]. Briefly, a total of 100 ng of total RNA was reverse transcribed to cDNA which was then used as template in an in-vitro transcription reaction followed by fragmentation of the single stranded cDNA and labeling through a terminal deoxy-transferase reaction. The biotinylated cDNA (5 µg) was fragmented and hybridized to an Affymetrix GeneChip Human Gene 1.0 ST Array with 764,885 probes representing 28,869 genes. GeneChips were then scanned using GeneChip Scanner 3000 7G Plus 2 and Command Console Software (AGCC) version 1.0 (Affymetrix Inc, Santa Clara, CA). Raw gene expression data in the generated CEL files were then normalized using the Robust MultiChip Averaging (RMA) algorithm [51] as implemented in Bioconductor [52]. To identify groups of functionally related genes that were differentially expressed, we conducted gene set analysis using the mixed effects models approach [23], [53]. The C5-BP collection of gene sets from http://www.broad.mit.edu/gsea/msigdb/index.jsp, which were derived from the controlled vocabulary of the Gene Ontology (GO) project, was used for these analyses. For each gene set, the mixed model included gene expression levels as the outcome variable, group (patients or healthy controls) as the fixed effects and different batches, patients as the random effects. In addition, random effects based on eigenvectors of the gene-gene correlation matrix were included to account for heterogeneous correlation patterns of the genes within a gene set [53]. Because many gene sets were examined, to control the rate of false positive findings by chance, we adjusted nominal p-values using the method of false discovery rate [54]. All microarray data is MIAME compliant and the raw data have been deposited in Gene Expression Omnibus (GEO) database, with the accession number GSE23832.

### Flow cytometry

PBMC were collected and incubated with CD4-FITC, CD8-APC and CD3-Pacific blue for 30 min on ice. After washing, the cells were fixed and permeabilized with AB reagents according to manufacturer's recommendation. PBMC were stained with p53-PE 30 min in the dark and analyzed on BD LSRII flow cytometer.

To detect apoptosis, lymphocytes were collected and stained with the Annexin V-FITC&7-AAD, as well as CD4-Pacific blue, CD8-APC and CD3-PE and signals were detected in BD LSRII flow cytometer. Data were analyzed by BD FACSDiva software and apoptosis was determined by the positive staining of annexin V and expressed as the increase in percentage of apoptotic cells in TSA treated samples when compared to cells treated with DMSO.

### Statistic analysis and software implementation

For the gene set analysis of microarray experiments, we used Procedure MIXED within the SAS software (version 8.1; SAS Institute, Cary, NC, USA) for fitting mixed effects models described in the section *Microarray Analysis*. Other results from real-time RT-PCR and flow cytometry were expressed as mean ± SD, and statistical comparisons between the groups were determined by T test using SPSS11.0 software and the R software (http://www.R-project.org).
